# Identification of *Trypanosoma brucei gambiense* in naturally infected dogs in Nigeria

**DOI:** 10.1186/s13071-019-3680-8

**Published:** 2019-08-27

**Authors:** Paschal Ugochukwu Umeakuana, Wendy Gibson, Romanus Chukwuduruo Ezeokonkwo, Boniface Maduka Anene

**Affiliations:** 1 Department of Veterinary Medicine, Faculty of Veterinary Medicine, University of Abuja, Abuja, Nigeria; 2 Department of Veterinary Medicine, Faculty of Veterinary Medicine, University of Nigeria, Nsukka, Nigeria; 3 School of Biological Sciences, University of Bristol, Bristol, BS8 1TQ UK; 4 Department of Veterinary Parasitology and Entomology, Faculty of Veterinary Medicine, University of Nigeria, Nsukka, Nigeria

**Keywords:** Canine trypanosomosis, *Trypanosoma brucei gambiense*, *Trypanosoma brucei brucei*, *Trypanosoma congolense*, Nsukka, Nigeria, Corneal opacity, *TGSGP*, *AnTat 11.17*

## Abstract

**Background:**

Animal trypanosomosis is endemic in Nigeria, while the human disease caused by *Trypanosoma brucei gambiense* is rarely reported nowadays after efforts to bring it under control in the 20th century. The University of Nigeria Veterinary Teaching Hospital (UNVTH) is a reference centre located within the Nsukka area and serves Enugu and neighboring states, Benue, Kogi, Anambra and Delta. Among dogs presented to the UNVTH with canine trypanosomosis, *T. brucei* is frequently reported as the causative agent. However, this is by morphological identification under the microscope, which does not allow distinction of human-infective (*T*. *b*. *gambiense*) and non-human-infective (*T. b. brucei*) subspecies. Here, we used subspecies-specific PCR tests to distinguish *T*. *b*. *gambiense* and *T. b. brucei*.

**Methods:**

Blood samples were collected on FTA cards from 19 dogs presenting with clinical signs of trypanosomosis at the UNVTH from January 2017 to December 2018. All dogs had a patent parasitaemia. DNA was extracted from the FTA cards using Chelex 100 resin and used as template for PCR.

**Results:**

All infections were initially identified as belonging to subgenus *Trypanozoon* using a generic PCR test based on the internal transcribed spacer 1 (ITS1) of the ribosomal RNA locus and a PCR test specific for the 177 bp satellite DNA of subgenus *Trypanozoon*. None of the samples were positive using a specific PCR test for *T. evansi* Type A kinetoplast DNA minicircles. Further PCR tests specific for *T*. *b*. *gambiense* based on the *TgsGP* and *AnTat 11.17* genes revealed that two of the dogs harboured *T*. *b*. *gambiense*. In addition to trypanosomes of subgenus *Trypanozoon*, *T. congolense* savannah was identified in one dog using a species-specific PCR test for this taxon.

**Conclusions:**

Nineteen dogs presenting with canine African trypanosomosis at UNVTH were infected with trypanosomes of the *T. brucei* group and in two cases the trypanosomes were further identified to subspecies *T. b. gambiense* using specific PCR tests. Thus *T. b. gambiense* is one of the parasites responsible for canine African trypanosomosis in the Nsukka area of Nigeria and represents a serious danger to human health.

## Background

Human African trypanosomosis (HAT), or sleeping sickness, is caused by protozoan parasites belonging to the *Trypanosoma brucei* complex in sub-Saharan Africa. The subspecies *Trypanosoma brucei gambiense* is the causative agent of the chronic form of the disease found in Central and West Africa, while *T. b. rhodesiense* is the agent of the virulent form in eastern and southern Africa. *Trypanosoma b. brucei* infects only domestic and wild animals [1]. *Trypanosoma b*. *gambiense* is divided into two sub-types or groups: the majority of isolates from human patients across the endemic region present a homogenous genetic composition, are avirulent in nature and belong to Group 1 *T*. *b*. *gambiense*, while a small minority identified predominantly in Côte d’Ivoire and Burkina Faso are genetically heterogeneous, show high virulence in experimental animals and belong to Group 2 [2–4].

Gambiense HAT caused by Group 1 *T*. *b*. *gambiense* (*Tbg*1) is considered to be an anthroponotic disease and consequently control programmes are generally aimed at stopping transmission by treating human cases and eliminating the tsetse vector [5]. However, animal reservoirs may be responsible for the endemic nature of HAT and its resurgence in the historic foci of West and Central Africa [5, 6]. *Tbg*1 has been isolated from pigs in Cameroon and Ivory Coast [2, 6–8], in sheep and goats in Cameroon, Equatorial Guinea and Congo [6, 9–11] and in pigs and a dog in Liberia [12]. Despite the identification of *Tbg*1 in various animals, there is an argument concerning their potential as animal reservoirs in sustaining *Tbg*1 transmission, based on the fact that these animals may not hold the disease for a long time. For example, dogs are considered to be sentinels for trypanosome infection rather than reservoir hosts, because dogs are very susceptible to trypanosome infection (*T. brucei* subspecies, *T. evansi*, *T. congolense*) and succumb rapidly, with death occurring within a few weeks without treatment [13]. In Kenya, outbreaks of *T*. *b. rhodesiense* in humans have been associated with outbreaks of blindness (corneal opacity) in dogs [14].

In the Nsukka area of Nigeria, *T. brucei* is highly prevalent in dogs [15] and also in pigs [16, 17], West African dwarf sheep and goats [18]. Tsetse flies (*Glossina tachinoides*) are abundant in the Nsukka area [19] and are found infected with trypanosomes (*T. brucei* and *T. congolense*) [19]. However, such reports relied on morphological identification by microscopy, which does not allow the distinction of different species and subspecies within subgenus *Trypanozoon*. Importantly, morphological identification fails to discriminate between human-infective and non-human-infective trypanosomes. The human serum resistance test, as originally devised by Rickman & Robson [20], was used to identify potentially human infective trypanosomes in one trade pig in Nsukka Area of Enugu State [21]. However, there has never been any report of human trypanosomosis in the Nsukka area of Enugu State, and HAT is not among the diseases commonly screened for by hospitals in Nigeria, even in areas where the tsetse vectors abound and trypanosomosis is reported in animals. In 2016, a case of HAT caused by *Tbg*1 was reported in a 58-year-old Nigerian woman visiting UK, who lived near Warri in Delta State, Nigeria [22]; according to the authors, no cases of HAT had been reported from Nigeria since 2012.

This study of dogs presenting with clinical signs of trypanosomosis at the UNVTH was conducted to determine which trypanosome species cause canine trypanosomosis in the Nsukka area of Nigeria and whether any dogs harbor the human-infective trypanosome, *Tbg*1.

## Methods

### Study population

Nsukka is located at 6°52ʹ–6°58ʹN, 7°20ʹ–7°27ʹE, covers an area of 1810 km^2^ and has a population of 309,633 [23]. The climatic conditions are characterized by high temperatures, averaging 27–28 °C. There are two seasons: the wet season extends from April to October, whilst the dry season extends from November to March. The annual rainfall range is 1680–1700 mm [24].

Blood samples were collected from 19 dogs presented to UNVTH for veterinary attention between 23rd January 2017 and 8th December 2018. On examination these dogs showed clinical signs of canine trypanosomosis including corneal opacity and enlarged lymph nodes, and were screened for trypanosomes by microscopy of wet blood smears. Demographic data, signalment (age, sex, breed and season) and clinical signs were recorded for each dog (Table 1). Blood samples from parasitologically-positive dogs were spotted on Whatman FTA cards, which were air-dried and stored in a cool, dry place until DNA extraction.

**Table 1:** Summary of patient data for 19 cases of Canine African Trypanosomosis presented at UNVTH

Case	Sex	Breed	Approx. age	LGA	Lymph node enlargement	Corneal opacity	Parasitaemia	PCV (%)	Outcome
1	M	Rottweiler	5 years	Nsukka	+	+	+++	22	Death
2	F	Mongrel	2 years	Nsukka	+	+	++	19	nk
3	–	–	–	–	−	−	++	nd	nk
4	F	Mongrel	2 years	Nsukka	+	+	+++	18	Death
5	F	–	4 years	Igbo-Eze North	+	+	+++	31	Death
6	F	Rottweiler	7 years	Nsukka	+	+	+++	nd	Death
7	M	Mastiff	2 years	Nsukka	+	-	++	28	Death
8	M	Rottweiler	–	Nsukka	+	-	+++	nd	Death
9	F	–	5 months	Udenu	+	+	+++	nd	nk
10	F	Mastiff	8 months	Nsukka	+	+	++	12	Death
11	F	Mongrel	2.5 years	Nsukka	+	+	++	nd	Recovery
12	–	–	–	Nsukka	+	+	+++	nd	nk
13	F	Caucasian	2 years	Udenu	+	+	+++	26	Death
14	M	–	9 months	Nsukka	+	+	+++	nd	Death
15	M	–	2 years	Nsukka	+	+	+	12	nk
16	F	Caucasian	6 months	Nsukka	+	+	+++	19	Death
17	M	Mongrel	2 years	Igbo-Eze South	−	−	+++	nd	nk
18	F	Mongrel	1.5 years	Nsukka	+	+	+++	20	Recovery
19	M	Mongrel	1.5 years	Nsukka	+	+	+++	nd	nk

*Abbreviations*: F, female; M, male; LGA, local government area; nd, not done; nk, not known

*Key*: +, <1 trypanosome/field; ++, 1–5 trypanosomes/field, +++, >5 trypanosomes/field

### DNA extraction

DNA was extracted from the FTA cards using Chelex 100 resin using a method adapted from [25]. Briefly, five 2-mm discs were removed from the center of each blood spot using a Harris Uni-Core disposable punch and washed twice in 1 ml of sterile distilled water for 10 min at room temperature with occasional vortexing. Samples were then centrifuged for 3 min at maximum speed (14,500× *rpm*) in a microcentrifuge and the water was removed. Two hundred microliters of a 5% w/v suspension of Chelex 100 resin in sterile water was added and samples were incubated at 56 °C for 20 min with vortexing every 10 min, followed by incubation at 95 °C for 10 min. The samples were vortexed, centrifuged as before for 5 min and 150 µl of the supernatant was then transferred to a clean microcentrifuge tube, being careful to avoid carrying over any Chelex 100 resin. DNA extracts were stored frozen at − 20 °C until use.

### Molecular identification by PCR

All PCRs were performed using DreamTaq polymerase (Thermo Fisher Scientific, UK) in 25 µl reaction volumes containing 5 µl of the template DNA and 0.4 µM primers (Table 2). Cycling conditions for ITS1 PCR were as specified by Adams et al. [26]; for other PCRs, cycling conditions were 95 °C for 3 min followed by 30 cycles of 95 °C for 45 s, × °C for 45 s and 72 °C for 45 s (where the annealing temperature × °C is specified in Table 2), ending with an extension reaction at 72 °C for 5 min. Positive and negative controls were included in each set of reactions: purified DNA of *T*. *b. brucei*, *T*. *b. gambiense* Group 1, *T*. *evansi* or *T. congolense* savannah, and water as negative control. Amplified products were resolved by electrophoresis through 1.7 % agarose gels and visualized by staining with ethidium bromide.

**Table 2:** PCR for detection of African trypanosomes

Target taxon/gene	Primer name	Primer sequence (5’–3’)	Annealing temperature (°C)	Amplicon size (bp)	Reference
*Trypanosoma* ITS1	TRYP3	TGCAATTATTGGTCGCGC	54	Various sizes according to species	[26]
	TRYP4	CTTTGCTGCGTTCTT			
Subgenus *Trypanozoon* 177-bp satellite repeat	TBR1	GAATATTAAACAATGCGCAG	60	164 (monomer)	[27, 32]
	TBR2	CCATTTATTAGCTTTGTTGC			
*T*. *congolense* savannah 350-bp satellite repeat	TCS1	CGAGAACGGGCACTTTGCGA	60	316 (monomer)	[27]
	TCS2	GGACAAACAAATCCCGCACA			
*T*. *evansi* Type A kDNA minicircle	EVA1	ACATATCAACAACGACAAAG	60	139	[33]
	EVA2	CCCTAGTATCTCCAATGAAT			
*T. b. gambiense* Group 1 *TgsGP* gene	TgsGP-F	GCTGCTGTGTTCGGAGAGC	50	308	[28]
	TgsGP-R	GCCATCGTGCTTGCCGCTC			
*T. b. gambiense* Group 1 *AnTat 11.17* VSG gene	AnTA-outer	CACAGACGACAGAAGCGATA	50	653	[29]
	AnTB-outer	GAAAGTGGGAGTTGTTGCTC			
	AnTC-inner	GCCTTCGAAGACACAAGCAG	50	381	
	AnTD-inner	XCGTCGTGCTGAAGTCTCCTG			

## Results

**Fig. 1:**
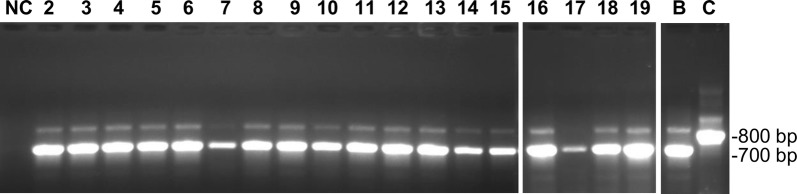
ITS1 PCR. Lane NC: water negative control; Lanes 2–19: blood samples from dogs (Table 1); Lane B: *T. b. brucei* J10; Lane C: *T. congolense* savannah WG81

Trypanosomes in the blood samples from the 19 dogs were initially identified by a generic PCR test based on the size of the ITS1 amplicon [26]; all 19 samples produced an amplicon of ~ 700 bp, consistent with identification as subgenus *Trypanozoon* (Fig. 1, Table 3). This result was confirmed using primers specific for the 177-bp satellite repeat of subgenus *Trypanozoon* (TBR1 and 2; Table 3).

**Table 3:** PCR results for 19 blood samples from dogs with canine trypanosomosis

Case	ITS1 (size in bp)	*Tz* satellite repeat	*Tcs* satellite repeat	*Tev* kDNA minicircle	*Tbg1 TgsGP*	*Tbg1 AnTat 11.17*	PCR ID
1	700	+	−	−	−	−	*Tbb*
2	700	+	−	−	+	−	*Tbg1*
3	700	+	−	−	−	−	*Tbb*
4	700	+	−	−	−	−	*Tbb*
5	700	+	−	−	−	−	*Tbb*
6	700	+	−	−	−	−	*Tbb*
7	700	+	−	−	−	−	*Tbb*
8	700	+	−	−	−	−	*Tbb*
9	700	+	−	−	−	−	*Tbb*
10	700	+	−	−	−	−	*Tbb*
11	700	+	−	−	−	−	*Tbb*
12	700	+	−	−	−	−	*Tbb*
13	700	+	+	−	−	−	*Tbb*, *Tcs*
14	700	+	−	−	−	−	*Tbb*
15	700	+	−	−	−	−	*Tbb*
16	700	+	−	−	−	−	*Tbb*
17	700	+	−	−	−	−	*Tbb*
18	700	+	−	−	−	−	*Tbb*
19	700	+	−	−	+	+	*Tbg1*

*Abbreviations*: ITS1, internal transcribed spacer; *Tz*, subgenus *Trypanozoon*; *Tcs*, *Trypanosoma congolense* savannah; *Tev*, *T. evansi*; *Tbg1*, *T. brucei gambiense* Group 1

*Key*: +, amplicon of expected size present, −, no amplicon present

No samples were identified as *T*. *evansi* using primers specific for the *T. evansi* Type A kinetoplast DNA minicircle (EVA1 and 2; Table 3). We conclude that all 19 dogs were infected with *T. brucei* and had therefore probably been infected by tsetse bite.

**Fig. 2:**
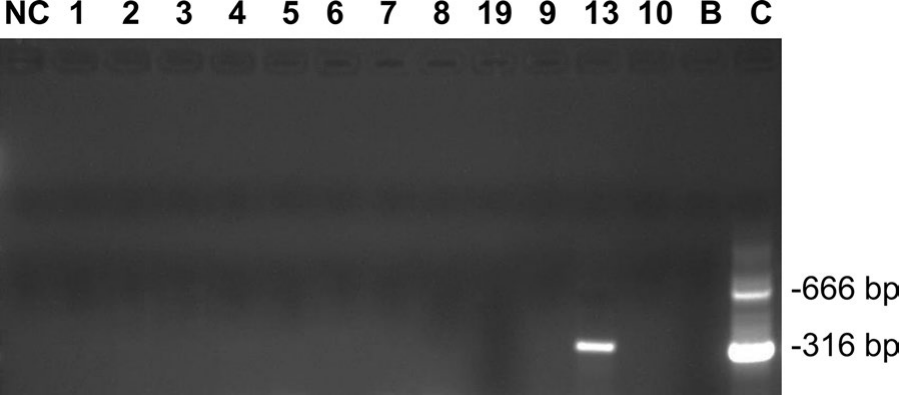
PCR specific for *Trypanosoma congolense* savannah. Lane NC, water negative control; Lanes 1–10, 13, 19: blood samples from dogs (Table 1, selected samples); Lane B: *T. b. brucei* J10; Lane C: *T. congolense* savannah WG81

As *T. congolense* had been identified in previous cases of canine trypanosomosis examined [15] (P. U. Umeakuana, unpublished), the 19 blood samples were also analysed by PCR specific for *T. congolense* savannah using primers targeted to the ~ 350-bp satellite DNA repeat [27]. One sample was positive (Fig. 2). As this sample had already been shown to be positive for *T. brucei* spp., this dog had a mixed infection. However, the expected ITS1 amplicon of ~ 800 bp for *T. congolense* savannah was not apparent (Fig. 1); we presume this is because trypanosomes of subgenus *Trypanozoon* were more numerous and/or the smaller 700-bp amplicon was preferentially amplified in the PCR reaction.

**Fig. 3:**
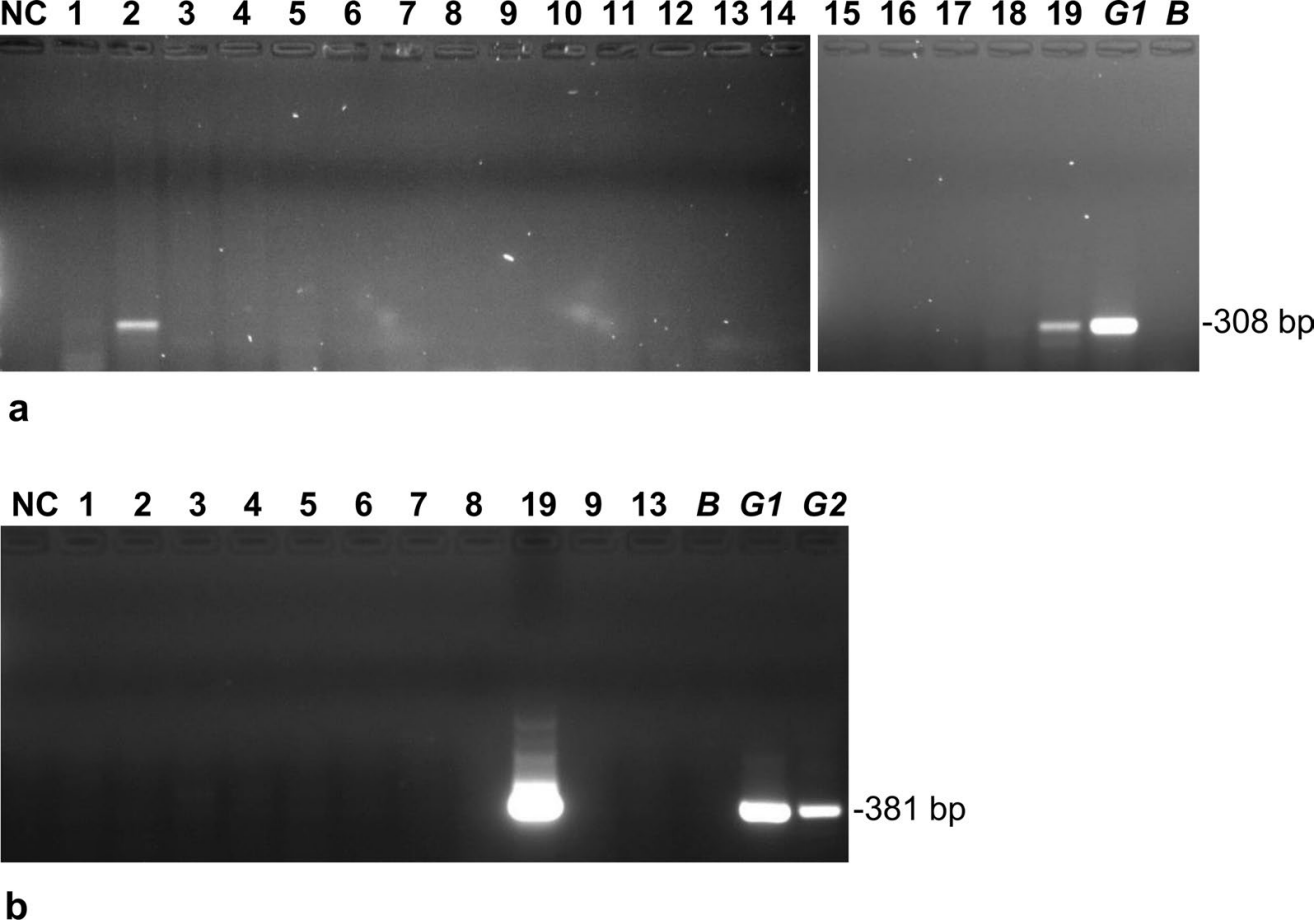
*Tbg1* specific PCR. **a** PCR amplification of the *TgsGP* gene; dog blood samples 2 and 19 are positive. **b** Nested PCR of the *AnTat11.17* VSG gene; sample 19 is positive. Lane NC: water negative control; Lanes 1–19: blood samples from dogs (Table 1); Lane B: *T. b. brucei* J10; Lane G1: *Tbg1* Bida 3; Lane G2: *Tbg1* NW2

To test whether any of the dogs were infected with the human pathogen *Tbg1*, two subspecies-specific PCRs were carried out using primers specific for the *TgsGP* gene [28] and the *AnTat 11.17* variant surface glycoprotein (VSG) gene, using a nested PCR [29]. Two of the 19 samples were positive for *TgsGP* and one was also confirmed to have the *Tbg1*-specific VSG gene *AnTat 11.17* (Fig. 3, Table 3). As the presence of the *TgsGP* gene is an unequivocal marker for *Tbg1* [28, 30], we conclude that two of the 19 dogs were infected with *Tbg1.* This may have been as the sole infection or mixed with *T. b. brucei*. The additional positive result for one sample with *AnTat 11.17* supports the identification of *Tbg1*. However, loss of VSG genes from the repertoire is not uncommon, so the absence of this gene in the other sample does not detract from its identification as *Tbg1*; indeed absence of *AnTat 11.17* in *Tbg1* has been reported previously [31].

## Discussion

All 19 dogs sampled in this study from the Nsukka area of Nigeria had canine trypanosomosis caused by trypanosomes of the *T. brucei* group, and in one case also *T. congolense* savannah. These dogs typically showed corneal opacity and were reported to have become blind by their owners. Several of the dogs were in extremely poor condition and died despite treatment with Diminazene aceturate. Most of the dogs had fever with temperatures of 40–42 °C and showed high parasitaemia with low PCV values. Anorexia, inappetence, unilateral and bilateral enlargement of superficial lymph nodes (popliteal, prescapular and submandibular lymph nodes) were common observations in the infected dogs. Other clinical aberrations observed were pale mucous membranes and evidence of loss of skin turgor.

Two of the dogs were shown to be infected with the human pathogen *T. b. gambiense* Group 1 (*Tbg1*) by subspecies-specific PCR tests. To the knowledge of the authors, no cases of HAT have been identified in the Nsukka area for the past 50 years, but the identification of two dogs harbouring the causative organism is worrying. Previously, a human serum resistant trypanosome was isolated from a trade pig in the Nsukka area [21]. Thus it is possible that HAT is endemic in the Nsukka area, but that sporadic cases of HAT have been misdiagnosed and gone unreported. Alternatively, the parasite may have been imported into the area through the movement of infected tsetse flies and/or animals. The Nsukka area is in Enugu State and shares a border with Benue State, in which one of the oldest HAT foci in Nigeria, i.e. Gboko, is located. Gboko neighbors the HAT endemic focus of Fontem in the Republic of Cameroon, which could make trans-boundary movement a possibility. The recently reported case from Nigeria [22] was from Warri in Delta State, which is approximately 225 km from Nsukka.

The epidemiological implications of our finding are controversial. Dogs have been adjudged to be sentinels of infection rather than reservoir hosts, because of their susceptibility to infection and the short course of disease, which is two to four weeks without treatment [13]. On the other hand, these are pet dogs harboring a dangerous human pathogen and living in close proximity to their owners and families. In addition, there is the possibility that other animals such as cattle, sheep, goats and pigs also have cryptic infection with the human pathogen. Thus, there is a need for systematic screening of livestock as well as dogs in this area to determine the level of prevalence of *Tbg1*. Importantly, human health practitioners in the area also need to be aware of the possibility of HAT in patients reporting with fever and/or other signs of trypanosome infection such as enlarged lymph glands and neurological problems.

## Conclusions

Nineteen dogs presenting with canine African trypanosomosis at UNVTH were infected with trypanosomes of the *T. brucei* group and in two cases the trypanosomes were further identified to subspecies *T. b. gambiense* using specific PCR tests. Thus *T. b. gambiense* is one of the parasites responsible for canine African trypanosomosis in the Nsukka area of Nigeria and represents a serious danger to human health.

## Data Availability

All data generated or analyzed during this study are included in this published article.

## Abbreviations

ITS1: internal transcribed spacer
PCR: polymerase chain reaction
*Tbg1*: *T. brucei gambiense* Group 1
*Tcs*: *Trypanosoma congolense* savannah
*Tev*: *T. evansi*
*Tz*: subgenus *Trypanozoon*
